# Ovarian Hyperstimulation Syndrome with pleural effusion: a case report

**DOI:** 10.1186/1757-1626-1-323

**Published:** 2008-11-18

**Authors:** Recep Yildizhan, Ertan Adali, Ali Kolusari, Mertihan Kurdoglu, Cagdas Ozgokce, Fulya Adali

**Affiliations:** 1 Department of Obstetrics and Gynecology, Yuzuncu Yil University, Van, Turkey; 2 Department of Radiology, Woman and Child Disease Hospital, Van, Turkey

## Abstract

**Background:**

To report a case of severe ovarian hyperstimulation syndrome (OHSS) with right pleural effusion following controlled ovarian hyperstimulation.

**Case presentation:**

A 24-year-old woman had severe OHSS as a complication of gonadotropin stimulation. The clinical picture showed enlarged ovaries, massive ascites, pleural effusion, abdominal pain, and dyspnea. Beside the medical treatment, abdominal paracentesis for the drainage of the massive ascites and tube thoracostomy were performed, resulting in expansion of the lung.

**Conclusion:**

Physicians can reduce the risk of OHSS by monitoring gonadotropin therapy and by withholding human chorionic gonadotropin medication. In in vitro fertilization protocols it can be advantageous to postpone the embryo transfer by freezing the embryos. Placement of a chest tube is a safe and efficient method for the treatment of pleural effusion.

## Background

Ovarian hyperstimulation syndrome (OHSS) is the most serious complication of controlled ovarian hyperstimulation. OHSS is cystic enlargement of the ovaries and a fluid shift from the intravascular space to the third space due to increased capillary permeability. Its occurrence is dependent on the administration of human chorionic gonadotropin (hCG) after an exaggerated ovarian response to gonadotropin stimulation. The syndrome is relatively common, occurring in up to 5% of women undergoing in vitro fertilization (IVF) or intrauterine insemination (IUI) procedures [1].

Despite close monitoring during ovarian stimulation and rigid guidelines and criteria for canceling cycles, OHSS still occurs. Clinical manifestations of OHSS can be classified into three forms. In mild forms of OHSS the ovaries are enlarged, while in moderate forms there is additional accumulation of ascites with mild abdominal distension. Its severe form in ~0.5% of stimulated cycles [2] is characterized by hemoconcentration, thrombosis, oliguria, pleural effusion, rarely pericardial effusion, and respiratory distress [3]. It can lead to life-threatening complications including thromboembolic events and even death.

## Case presentation

**Figure 1:**
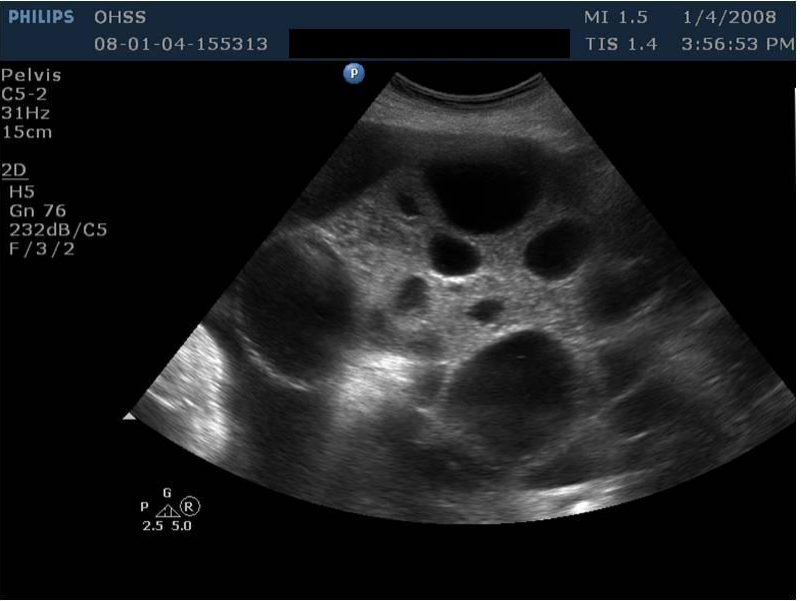
Ultrasonographic examination revealed bilaterally enlarged multicystic ovaries.

A 24-year-old woman was hospitalized in a state hospital because of confirmed bilateral ovarian cysts and ascites. Then she was referred to our hospital because of increasing abdominal girth and dyspnea of 1 week's duration. The patient's weight was 80 kg and height was 162 cm. The stimulation was performed by an external unit using a long protocol with follitropin alfa. The stimulation cycle had to be discontinued because of OHSS. The patient showed a severe form of OHSS with massive ascites, abdominal pain, dyspnea, blood pressure 80/40 mmHg, pulse rate 100 beats/min, plasma estradiol 4000 pg/mL, β-hCG 86 IU/L, hemoconcentration of 52%, hemoglobin of 18.0 g/dL, leucocytosis of 29 × 10^3^/μL, PaO_2 _of 70 mmHg, PaCO_2 _of 40 mmHg, pH of 7.44, and oxygen saturation of 91.2%. The ultrasonographic examination revealed bilaterally enlarged multicystic ovaries and a large amount of ascites (Figure 1).

**Figure 2:**
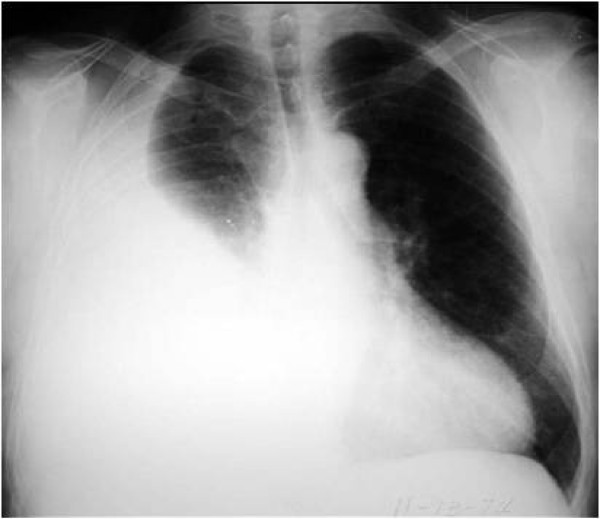
The anteroposterior chest X-ray revealed right pleural effusion.

Immediately after admission, infusion therapy was started, consisting of normal saline-infusion 0.9% 1000 ml (Pharmacia), glucose 5% 1000 ml (Pharmacia), four times 50 ml of human serum albumin 20%, and low-molecular weight heparin 5000 IU 2 × 1 per day. Body weight, abdominal circumference, intake and outputs, ultrasonography, and laboratory studies were monitored strictly daily. Renal function was supported using diuretics (Furosemide 20 mg ampoule and Furosemide tablet). Renal function was not disturbed and there were no serious changes in serum electrolytes. The patients' girth increased between days 2 and 7, and then abdominal paracentesis for the drainage of the massive ascites became necessary. The patient reported increasing dyspnea. The anteroposterior chest X-ray (Figure 2) revealed right pleural effusion, whereupon a chest-tube was placed for treatment of pleural effusion.

## Discussion

Ovarian hyperstimulation syndrome (OHSS) is an iatrogenic, serious complication of controlled ovarian hyperstimulation, usually self-limited, but occasionally life threatening and typically occurs with gonadotropin, rarely with clomiphene citrate [4]. Without hCG, OHSS is extremely rare. The symptoms are more severe and persist longer if pregnancy is successful. Patients who are pregnant sustain the ovarian luteinization process by the production of hCG. Although the pathophysiology of this syndrome has not been completely elucidated, the underlying mechanism responsible for the clinical manifestations of OHSS appears to be an increase in capillary permeability of mesothelial surfaces [5]. There is increasing evidence that certain vasoactive substances such as vascular endothelial growth factor (VEGF), cytokines (IL-2, IL-6, and IL-8), tumor necrosis factor-alpha (TNF-alpha), and the ovarian renin-angiotensin system, which are activated by gonadotropin, can lead to increased vascular permeability and extravascular fluid accumulation in OHSS [6,7].

VEGF has a major role in the pathogenesis of OHSS. VEGF is a heparin-binding glycoprotein with vascular permeability-enhancing, angiogenic, and endothelial cell-specific mitogenic activities [8]. VEGF increases vascular permeability, which may explain fluid leakage in the third space. This leakage is responsible for the development of ascites, pleural effusions, edema, and hemoconcentration [9]. Clinical characteristics of OHSS include ascites and pleural effusion induced by increased vascular permeability, where VEGF was suspected to be the culprit. Through up-regulation of VEGF, hCG plays a significant role in the pathogenesis of OHSS [10]. Navot et al. [11] detected high levels of prorenin and angiotensin 2 in patients' follicle fluid and high renin concentrations in their plasma.

OHSS, characterized by third space fluid shift and intravascular volume depletion, results in massive ascites and hydrothorax. There are various comments about the development of the pleural fluid. It is adduced that high estrogen levels cause pleural effusion. Pleural effusion usually occurs in the severe forms of OHSS [12]. In our case pleural effusion occurred during the initial days of treatment. Loret de Mola et al [13] observed that pleural effusion in OHSS usually occurs on the right side of the lung, as in our case. They offer the explanation that lymphatic drainage on the right is less than that on the left and the diaphragmatic hollows are greater on the right. It is possible that pleural effusion originates from the fluid shift from abdominal ascites [12].

From the outset of treatment, our patient, who had severe dyspnea and impaired blood gases, was protected from possible pulmonary emboli. We gave a light molecular heparin. Then we eliminated the pulmonary emboli. Thoracocentesis is safe and efficient for the treatment of hydrothorax and lung collapse in cases of OHSS and may be repeated as often as necessary [14]. Our patient also required either paracentesis or thoracentesis four times. A chest-tube was installed in the patient by thoracic surgeons.

If the OHSS develops within an IVF protocol, it may be prudent to postpone the embryo transfer since the establishment of pregnancy can lengthen the recovery time or contribute to a more severe course. Instead of canceling the cycle, it is also possible to administer HCG, to retrieve the oocytes, and to freeze all embryos.

Physicians can reduce the risk of OHSS by monitoring FSH therapy to use this medication cautiously, and by withholding hCG medication. The patients with OHSS must be treated urgently and with multidisciplinary management. If left untreated, OHSS can result in serious health complications and even death.

## Consent

Written informed consent was obtained from the patient for publication of this case report and accompanying images. A copy of the written consent is available for review by the Editor-in-Chief of this journal.

## Competing interests

The authors declare that they have no competing interests.

## Authors' contributions

All authors were involved in patient's care. RY, EA, AK prepared the manuscript, MK, CO, FA edit and coordinated the manuscript. All authors read and approved the final manuscript.
